# Editorial: Tuberculosis and Non-tuberculous Mycobacteria Infections: Control, Diagnosis and Treatment

**DOI:** 10.3389/fpubh.2021.666187

**Published:** 2021-10-28

**Authors:** Onya Opota, Jesica Mazza-Stalder, Miguel Viveiros, Emmanuelle Cambau, Miguel Santin, Delia Goletti

**Affiliations:** ^1^Institute of Microbiology, University of Lausanne and Lausanne University Hospital, Lausanne, Switzerland; ^2^Respiratory Medicine Department, University of Lausanne and Lausanne University Hospital, Lausanne, Switzerland; ^3^Global Health and Tropical Medicine, GHTM, Instituto de Higiene e Medicina Tropical, IHMT, Universidade Nova de Lisboa, UNL, Lisbon, Portugal; ^4^UMR1147 IAME, Inserm, Université de Paris, APHP-GHU Nord, Service de Mycobactériologie Spécialisée et de Référence, Centre National de Référence des Mycobactéries et de la Résistance des Mycobactéries aux Antituberculeux (CNR-MyRMA), Paris, France; ^5^Service of Infectious Diseases, Tuberculosis Unit, Bellvitge University Hospital-IDIBELL, University of Barcelona, Barcelona, Spain; ^6^Translational Research Unit, National Institute for Infectious Diseases L. Spallanzani-IRCCS, Rome, Italy

**Keywords:** *Mycobacterium tuberculosis*, tuberculosis, multidrug resistant, whole genome sequencing, tuberculosis treatment, minimal inhibition concentration, microbiota, non-tuberculous mycobacteria

Tuberculosis (TB), is one of the top 10 causes of death worldwide (WHO). According to the last Global TB report from the World Health Organization, 10 million persons were estimated to have had TB in 2019 worldwide, causing about 1.6 million deaths. Tuberculosis has not only a dramatic impact on the quality of life for the patients, but also has raised many socio-economic issues at a community level, especially in medium and high burden regions, such as India, China, and Indonesia.

In 2014, WHO adopted the “End TB strategy” which aimed to reduce TB deaths by 90% between 2015 and 2030, to prevent new cases by 80% during the same period and to decrease the socio-economic impact of the disease at a family level. Even though tuberculosis global incidence has decreased significantly, efforts still need to be made to reach these goals.

Non-tuberculous mycobacteria (NTM), in contrast to *Mycobacterium tuberculosis*, are bacteria widely spread in the environment and can be found in a broad range of ecosystems such as soils and water, including drinking water systems. NTM are opportunistic pathogens associated with both pulmonary and extrapulmonary infections.

This Research Topic collected articles addressing: (i) TB and NTMs associated diseases, diagnostic, control, and public health, (ii) mycobacterial genomics, (iii) and antimycobacterial drugs and resistance.

Efforts have been made to present the latest technical and scientific approaches for resistance prediction and mutation ranking in *M. tuberculosis*. This included the use of multi-label random forest models from whole genome sequences to rank and identify important mutations for better prediction of first-line drugs resistance (Kouchaki et al.). According to the major advances achieved in the last years, diagnostic and epidemiological approaches based on genomics have dramatically increased. Whole genome sequencing (WGS) is widely used to identify clusters of transmission of *M. tuberculosis* in different settings like in Ghana, where WGS analysis was used to resolve clusters and explored the spatial distribution of confirmed recent transmission events (Asare et al.). WGS was useful in detecting unsuspected outbreaks as well as to better understand the dynamic of *M. tuberculosis* lineages (Outhred et al.). Different approaches are now available for rapid and accurate drug susceptibility testing including phenotypic and molecular methods based on DNA sequencing. This latter approach has shown its added value for early identification of resistance to anti-TB drugs, including ethambutol (Li et al.; Wan et al.).

Finally, the study of *M. tuberculosis* wild-type MIC distributions addressed the possibility of re-evaluating the critical concentration of anti-TB drugs and pharmacodynamics or optimizing the drug dosage (Dusthackeer et al.). In the endeavor of TB treatment and anti-TB drugs discovery, a new competitive inhibitor of D-alanine–D-alanine ligase A (DdlA) (IMB-0283) that is more potent than the classical D-cycloserine (DCS) has been identified *via* high-throughput screening (Meng et al.). The MIC of IMB-0283 for the standard and clinical drug-resistant *M. tuberculosis* strains ranged from 0.25 to 4.00 μg/mL, whereas that of DCS was 16 μg/mL. This new inhibitor revealed to have lower cytotoxicity and to be more efficacious *in vivo* than DCS, a still important second line antituberculosis drug. Alongside, and in view of the urgent need of more efficacious and less toxic second line antituberculosis drugs, the potential of the recently described cephalosporins, selectively active against non-replicating *Mycobacterium tuberculosis* forms, has been reported. Using alkyne analogs of these cephalosporins and an activity-based protein profiling, over 30 new protein binders were identified related to mycobacterial survival in a non-replicative state and inhibiting the cell by collective action on multiple targets (Lopez Quezada et al.). Improvement of anti-TB regimen, including regimen for the treatment of multidrug-resistant tuberculosis is paramount as such treatment have been shown to have long-term effects on gut microbiota (Wang et al.).

Regarding the treatment of NTM infections, a retrospective analysis focusing on *Mycobacterium abscessus* pulmonary infection reported that among 244 patients, only 110 patients met the criteria for treatment, and the outcomes of treatment were mostly unsatisfactory, particularly among patients suffering from *Mycobacterium abscessus subsp. abscessus* lung disease (Chen et al.). The administration of amikacin, imipenem, linezolid, and tigecycline correlated with increased treatment success. Genomics were also now established as a powerful tool to refine and complete the systematics and classification of NTMs as shown for the *M. kansasii* complex and subtypes reclassified using a three-pronged computational strategy based on the alignment fraction-average nucleotide identity, genome-to-genome distance, and core-genome phylogeny combined with five canonical taxonomic markers (16S rRNA, *hsp*65, *rpo*B, *tuf* genes, and 16S-23S rRNA intergenic spacer region; Jagielski et al.). A study on another clinical important NTM, the *M. avium* complex (MAC) determined the impact of using two different culture media used for MAC drug susceptibility testing. MIC determination using the microdilution method for antibiotics used in the treatment of MAC infections has a clear clinical relevance and accuracy for determining the resistance levels of this important group of NTM (Jaffré et al.). The raise of the medical importance of NTM due to the increasing number of immune-compromised hosts (solid organ transplant recipients and oncologic patients among others) highlighted the importance of a better understanding of the source of contamination and measures to reduce exposure to these sources (Norton et al.; Lecorche et al.).

Major advances have been made in the field of TB and NTM relying on many new technological opportunities, new approaches and new paradigms. This led to a better understanding of these pathogens as well as in improvement of patients care and disease control. Regarding patients care, the importance of multidisciplinary approaches taking into account medico-socio-economic considerations was highlighted (Mazza-Stalder et al.; Pujol-Cruells and Vilaplana). Interestingly, this Research Topic also highlighted the inhomogeneity of practices among Europe for the management of TB (Méchaï et al.). Efforts need to be intensified in parallel with increased translational collaboration to guarantee the application and rapid sharing of new discoveries.

## Author Contributions

OO, JM-S, MV, EC, MS, and DG contributed to the redaction of the manuscript. All authors contributed to the article and approved the submitted version.

## Conflict of Interest

The authors declare that the research was conducted in the absence of any commercial or financial relationships that could be construed as a potential conflict of interest.

## Publisher's Note

All claims expressed in this article are solely those of the authors and do not necessarily represent those of their affiliated organizations, or those of the publisher, the editors and the reviewers. Any product that may be evaluated in this article, or claim that may be made by its manufacturer, is not guaranteed or endorsed by the publisher.

